# Supplementary material for: Polyketide synthase gene clusters in the ericoid mycorrhizal fungus Oidiodendron maius: genome-wide prediction, expression profiling, and functional characterization of knockout mutants
Source: Front Plant Sci. 2026 Sep 16;17:1924430. doi: 10.3389/fpls.2026.1924430 (PMC13623903; doi:10.3389/fpls.2026.1924430)
Supplement: Supplementary file 1 [file SupplementaryFile1.zip › Supplementary Materials/Supplementary Folder 1.zip!/Oidma1_AssemblyScaffolds_Repeatmasked/knownclusterblast/cluster35/jgi.p_Oidma1_65474_mibig_hits.html]

| MIBiG Protein | Description | MIBiG Cluster | MiBiG Product | % ID | % Coverage | BLAST Score | E-value |
| --- | --- | --- | --- | --- | --- | --- | --- |
| AEN83885.1 | AdaR | BGC0000156 | TAN-1612\_biosynthetic\_gene\_cluster | 51 | 101.564945227 | 583 | 2.6e-166 |
| EGD99352.1 | hypothetical\_protein | BGC0001144 | Neosartoricin\_biosynthetic\_gene\_cluster | 42 | 85.289514867 | 411 | 2.3e-114 |
| ADI24939.1 | VrtR2 | BGC0000168 | Viridicatumtoxin\_biosynthetic\_gene\_cluster | 45 | 82.0031298905 | 396 | 5.8e-110 |
